# Evaluation of Anti-Inflammatory Components of Guizhi Fuling Capsule, an Ancient Chinese Herbal Formula, in Human Umbilical Vein Endothelial Cells

**DOI:** 10.1155/2020/2029134

**Published:** 2020-10-21

**Authors:** Yuzhong Zheng, Guizhong Xin, Guowei Gong, Tina TX Dong, Ping Li, Karl W. K. Tsim

**Affiliations:** ^1^School of Life Sciences and Food Technology, Hanshan Normal University, Chaozhou, Guangdong 521041, China; ^2^Division of Life Science and Center for Chinese Medicine, The Hong Kong University of Science and Technology, Clear Water Bay Road, Hong Kong, China; ^3^State Key Laboratory of Natural Medicines, China Pharmaceutical University, Nanjing 210009, China; ^4^Department of Bioengineering, Zunyi Medical University, Zhuhai Campus, Zhuhai, Guangdong 519041, China

## Abstract

**Background:**

Guizhi Fuling capsule (GFC), a well-known formula composed of five medicinal herbs, is commonly prescribed to treat primary dysmenorrhea, as well as to achieve good clinical efficacy in China. However, the active components of GFC have not been identified. Here, the anti-inflammatory functions of GFC, as well as its major ingredients, were evaluated in human umbilical vein endothelial cells (HUVECs).

**Methods:**

Lipopolysaccharide (LPS) was used in HUVECs to imitate the cellular inflammation. Then, GFC-triggered mRNA expressions of cyclooxygenase-1 (COX-1) and COX-2 were determined by real-time PCR, while the expression of COX-2 protein was revealed by western blotting. Besides, nine components of GFC were evaluated for their contribution value in the anti-dysmenorrhea effects

**Results:**

The application of GFC downregulated the mRNA expressions of COX-1 and COX-2 mRNAs. Nine major components of GFC were tested in the inflammatory system, and three compounds, including paeoniflorin, benzoylpaeoniflorin, and amygdalin, exhibited robust activation in HUVECs. The combination of paeoniflorin, benzoylpaeoniflorin, and amygdalin showed over 80% of the anti-inflammatory activation.

**Conclusion:**

Our study supports that GFC plays a promising role in anti-dysmenorrhea function by decreasing COXs' expression. Besides, paeoniflorin, benzoylpaeoniflorin, and amygdalin could be considered as major regulators for the anti-dysmenorrhea effects of GFC.

## 1. Background

Dysmenorrhea, defined as difficult menstrual flow or painful menstruation, is one of the most common gynecologic complaints in young women who present to clinicians [1]. Aetiological studies display that the release of uterine prostaglandins could stimulate uterine contractions and ischemia leading to pain [2]. Prostaglandins generated from arachidonic acid are under the action of cyclooxygenase (COX) [3]. Among the isoforms of COX, COX-1 is constitutively expressed in the cells and is involved in homeostasis, whereas COX-2 is induced in response to pain and inflammation [3]. The mechanism of dysmenorrhea during menstruation is highly related to COX-2 expression, leading to an increase in prostaglandins [4]. Until now, the main drug used to treat dysmenorrhea has been nonsteroidal anti-inflammatory drugs, but may cause side effects, including problems in the digestive tract, liver, and kidney [5].

As a well-known traditional Chinese medicine (TCM) formula, Guizhi Fuling capsule (GFC) originated from Jingui Yaolue (220 AD) by Zhang Zhongjing. GFC consists of five herbs at the same proportion: *Cinnamomum cassia* Presl (Cinnamomi Ramulus), *Poria cocos* (Schw.) Wolf (Poria), *Paeonia suffruticosa* Andr. (Moutan Cortex), *Paeonia lactiflora* Pall. (Paeoniae Radix Alba), and *Prunus persica* (L.) Batsch (Persicae Semen) (Chinese Pharmacopoeia, 2020). GFC, as a prescription drug, has been widely used to treat blood stasis syndromes in gynecology diseases, e.g., primary dysmenorrhea and endometriosis in China [6–8]. However, the anti-inflammatory process and active components of GFC have not been clearly elucidated. Therefore, the present study aimed to elucidate the anti-dysmenorrheal effects of GFC by determining its effects on human umbilical vein endothelial cells (HUVECs). Besides, anti-inflammatory components of GFC were identified and their efficacy contribution was evaluated.

## 2. Methods

### 2.1. Reagents and Materials

The GFC samples were purchased from Jiangsu Kanion Pharmaceutical Co., Ltd. (Lianyungang, China). The voucher specimens of GFC were deposited in the Centre for Chinese Medicine of HKUST. The powder in the GCF was taken out and then dissolved in ddH_2_O. The GFC solutions were filtered using a 0.22-micron filter at 100 mg/mL, and the concentration was then confirmed by weighing the dry matter in solutions.

Paeoniflorin (>99%), benzoylpaeoniflorin (>99%), amygdalin (>99%), paeonol (>99%), cinnamic aldehyde (>99%), cinnamic acid (>99%), pachymic acid (>99%), gallic acid (>99%), and benzoic acid (>99%) were obtained from the National Institute for the Control of Pharmaceutical and Biological Products (Beijing, China). All chemicals were dissolved in DMSO at 1 mg/mL as stock solutions.

3-(4,5-Dimethylthiazol-2-yl)-2,5-diphenyl tetrazolium bromide (MTT), A23187, L-NAME, and BAPTA-AM (all at >98% purity) were purchased from Sigma Chemical Co. (St. Louis, MO). LY294002 was purchased from Cell Signaling Technologies (Danvers, MA). Ultrapure water was prepared from a Milli-Q purification system (Millipore, Molsheim, France). COX-2 antibody (sc1745) was purchased from Santa Cruz Biotechnology (USA). GAPDH rabbit mAb (5174S) was purchased from Cell Signaling Technology (Danvers, MA). Goat anti-rabbit IgG-HRP (sc2004) was purchased from Santa Cruz Biotechnology. All culture reagents were from Invitrogen (Carlsbad, CA). All chemicals used were of AR grade or HPLC grade.

### 2.2. Cell Culture

HUVEC cells, purchased from American Type Culture Collection (ATCC, Manassas, VA), were cultured on a 0.2% gelatin-coated T75 flask maintained in a culturing medium (M199) at 37°C in a water-saturated 5% CO_2_ incubator. The culture medium was composed of 20% fetal bovine serum, 90 mg/mL heparin sodium salt, 20 *μ*g/mL endothelial cell growth serum (ECGS), 100 U/mL penicillin, and 100 *μ*g/mL streptomycin. The passage 3 to 8 of HUVEC cells was used in these studies to ensure the genetic stability of the culture [9].

### 2.3. Viability Assay of Cells

HUVEC cells were cultured and stabilized in a 96-well plate for 24 h. The series of concentrations of GFC or other chemicals were used to treat the cells for another 24 h. Then, the cell viability test was performed with 0.5 mg/mL of MTT in PBS for 3 h. After the solution was removed, the cell lysis was resuspended in DMSO and then measured at 570 nm absorbance. Finally, the cell viability was calculated based on the control.

### 2.4. Quantitative Real-Time PCR

After treating with GFC or chemicals, HUVEC cells were collected to analyze the mRNA expression of COX-1 and COX-2. According to the manufacturer's instructions (Invitrogen), total RNA of HUVECs was isolated by using the TRIzol reagent and reverse transcribed into cDNAs. The mRNA expression was analyzed by real-time PCR with using the SYBR Green Master mix and ROX reference dye according to the manufacturer's instructions (Applied Bioscience, Foster City, CA). The optimized primers were as follows: 5′-AG GAG ATG GCA GCA GAG TT-3′ and 5′-GTG GCC GTC TTG ACA ATG TT-3′ for human COX-1 (236 bp; NM_001271166.1); 5′-TGA ATG GGG TGA TGA GCA GT-3′ and 5′-GGG ATG CCA GTG ATA GAG GG-3′ for murine COX-2 (205 bp; NM_000963.3). As an internal control, GAPDH primers were 5′-CTT CCC GTT CAG CTC TGG G-3′ and 5′-AAC GGA TTT GGC CGT ATT GG-3′ (657 bp; NR_0215885). The SYBR Green signal was detected using the Mx3000P™ Real-Time System (Agilent, Stratagene). PCR reactions were performed in triplicate for each sample, and the expression levels were normalized to a GAPDH gene. To confirm the specific amplification of PCR products, gel electrophoresis and melting curve analysis was performed.

### 2.5. Determination of COX-2 Protein

In 6-well plates, HUVEC cells were treated by drugs (including activators and blockers) for 48 h. The cultures were harvested with high salt lysis buffer and centrifuged at 16,100 rpm for 10 min at 4°C. The protein was collected and subjected to SDS-PAGE analysis. The membrane-transferred proteins were incubated with anti-COX-2 antibodies (Cell Signaling) at 1 : 1,000 dilution at 4°C for 12 h. Following incubation in horseradish peroxidase- (HRP-) conjugated anti-rabbit secondary antibodies in 1 : 5,000 dilutions for 3 h at 25°C, the immune complexes were visualized by the enhanced ECL method (Amersham Biosciences). An image analyzer was used to compare the band intensities in the control and agonist-stimulated samples on the same gel.

### 2.6. Statistical Analysis and Other Assays

Concentrations of protein were tested by Bradford's method (Hercules, CA). Statistical tests were performed by using one-way analysis of variance. Data were expressed as mean ± SEM, *n* = 4 − 5. Statistically significant changes were classified as significant (^*∗*^) where *p* < 0.05, more significant (^*∗∗*^) where *p* < 0.01, and highly significant (^*∗∗∗*^) where *p* < 0.001.

The Minimum Standards of Reporting Checklist contains details of the experimental design, statistics, and resources used in this study.

## 3. Results

### 3.1. GFC Downregulates Cyclooxygenase Expression

**Figure 1:**
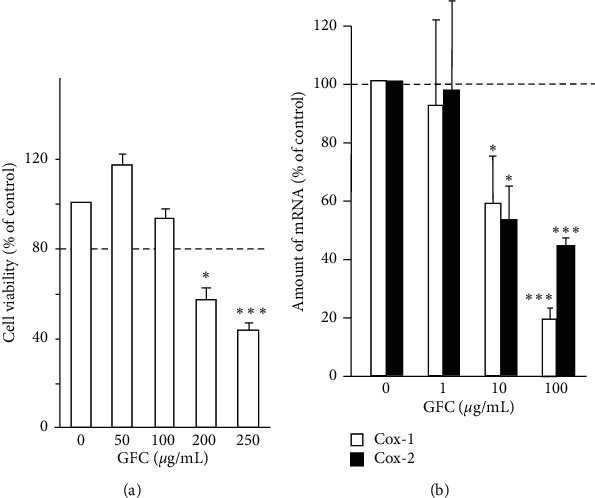
GFC downregulates cyclooxygenase expression in HUVECs. (a) Different amounts of GFC (0–250 *μ*g/mL) were applied onto cultured HUVEC cells for 48 hours, and then the cell viability was determined. (b) HUVEC cells were treated with GFC (0–100 *μ*g/mL) for 48 hours. Total RNAs were extracted and reverse transcribed to cDNA for real-time PCR analysis. The mRNA levels of COX-1 and COX-2 were determined by the Ct-value method and normalized by the mRNA level of a house-keeping gene GAPDH. Values were expressed as the percentage of control (untreated culture). Data were expressed as mean ± SEM, where *n* = 4; ^*∗*^*p* < 0.05; ^*∗∗∗*^*p* < 0.001.

Abnormal upregulation of COXs, especially COX-2, is commonly involved in several inflammation-induced diseases, e.g., dysmenorrhea [3,4]. We determined the inhibitory effects of GFC on the expression of COX-1 and COX-2 in HUVECs. Firstly, the application of GFC in HUVECs did not affect the cell viability when its dosage was lower than 100 *μ*g/mL (Figure 1(a)), which decided a dosage range of GFC for the following experiments. GFC from 1 to 100 *μ*g/mL was applied onto the HUVEC culture for 48 hours, which downregulated COX (COX-1 and COX-2) expressions in a dose-dependent manner. 100 *μ*g/mL of GFC showed a maximal effect of ∼80% and 60% inhibition to COX-1 and COX-2, respectively (Figure 1(b)).

**Figure 2:**
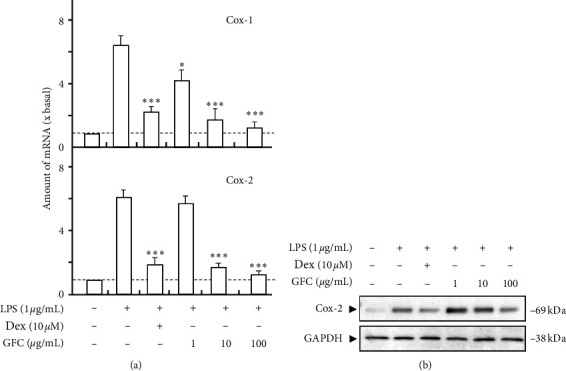
GFC inhibits LPS-induced cyclooxygenase expression. (a) Cultured HUVEC cells were pretreated with LPS (1 *μ*g/mL) for 3 hours before the application of different GFC (0–100 *μ*g/mL) for 48 hours. The mRNA levels of COX-1 and COX-2 were determined by the Ct-value method and were normalized by the mRNA level of GAPDH. (b) The cell lysates were collected to determine the protein expressions of COX-2 (∼69 kDa) using a specific antibody. Ten *μ*M of dexamethasone (Dex) served as positive control. GAPDH (∼38 kDa) served as loading control. Quantification of protein amount from the blot was calculated by using a densitometer. Values were expressed as the fold of increase to basal reading (untreated culture). Data were expressed as mean ± SEM, where *n* = 4; ^*∗*^*p* < 0.05; ^*∗∗∗*^*p* < 0.001.

In cultured HUVECs, application of lipopolysaccharide (LPS), an activator of inflammation, induced the expressions of COX-1 and COX-2 to over 6-folds (Figure 2(a)). Dexamethasone, served as a positive control of immune suppressor, sharply reduced COX-1 and COX-2 expressions to ∼2-fold of the control. When the cells were treated with GFC for 48 h after applying LPS, the mRNA expressions of COX-1 and COX-2 were inhibited in a dose-dependent manner. 100 *μ*g/mL of GFC could obtain maximal inhibition, which decreased by 80% (Figure 2(a)). Regarding LPS-induced COX expression, the suppression of GFC was better than that of dexamethasone.

In addition, LPS also induced COX-2 protein expression in HUVECs. This induction could be suppressed by GFC. In the background, the COX-2 protein (∼69 kDa) was barely detectable in HUVECs. With the treatment of LPS, the expression of COX-2 protein increased dramatically (Figure 2(b)). However, the addition of GFC could significantly reduce the LPS-induced COX-2 protein expression, as in the case of mRNA expression. Moreover, the suppression of COX-2 expression by GFC was in a dose-dependent manner. Dexamethasone served as the positive control (Figure 2(b)).

### 3.2. Compounds of GFC Inhibits LPS-Induced Cyclooxygenase Expression

GFC was provided by Jiangsu Kanion Pharmaceutical Co., Ltd. We selected nine chemicals to qualify GFC, i.e., paeoniflorin, benzoylpaeoniflorin, amygdalin, paeonol, cinnamic aldehyde, cinnamic acid, pachymic acid, gallic acid, and benzoic acid. The chemicals are supposed to be active ingredients, and their contents in GFC have been summarized in Table 1, according to previous publication [7, 10]. Among them, the chemical contents of top four were paeoniflorin, amygdalin, paeonol, and gallic acid; they were more than 0.8% in GFC. The quality of GFC exceeded the requirements as stated in Chinese Pharmacopoeia (2020).

In addition, nine selected chemicals were evaluated with the biological analyses. The cytotoxicity of all chemicals in cultures was tested. The suitable doses of two chemicals, cinnamic aldehyde and pachymic acid, were lower than 20 *μ*M and 25 *μ*M, respectively. The other chemicals did not affect the cell viability when their dosages were lower than 250 *μ*M (Table 1). At the same time, the inflammatory response of all chemicals was evaluated in HUVECs. Unexpectedly, three chemicals, paeoniflorin, benzoylpaeoniflorin, and amygdalin, exhibited potential bioactivity to inhibit COX expressions. The others did not show suppression responses (data not shown). Comparing the response value of three compounds in HUVECs, the active strong-to-weak sequence was paeoniflorin > benzoylpaeoniflorin > amygdalin.

**Figure 3:**
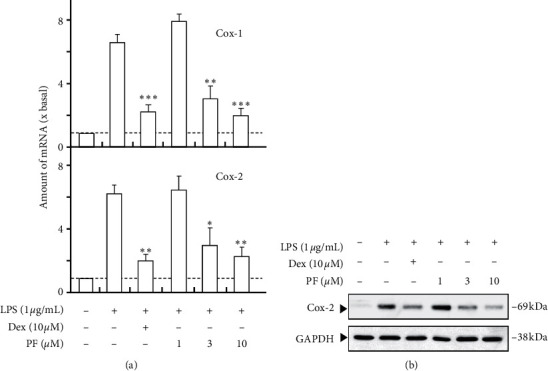
Paeoniflorin inhibits LPS-induced cyclooxygenase expression. (a) Cultured HUVEC cells were pretreated with LPS (1 *μ*g/mL) for 3 hours before the application of paeoniflorin (PF; 0–10 *μ*M) for 48 hours. The mRNA levels of COX-1 and COX-2 were determined by the Ct-value method and normalized by the mRNA level of GAPDH. (b) The cell lysates were collected to determine the protein expressions of COX-2 using a specific antibody. Ten *μ*M of dexamethasone (Dex) served as positive control. GAPDH served as loading control. Quantification of protein amount from the blot was calculated by a densitometer. Values were expressed as the fold of increase to basal reading (untreated culture). Data were expressed as mean ± SEM, where *n* = 4. ^*∗*^*p* < 0.05; ^*∗∗*^*p* < 0.01; ^*∗∗∗*^*p* < 0.001.

**Figure 4:**
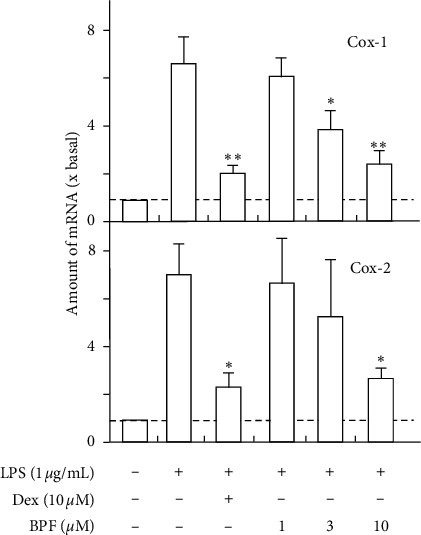
Benzoylpaeoniflorin inhibits LPS-induced cyclooxygenase expression. Cultured HUVEC cells were pretreated with LPS (1 *μ*g/mL) for 3 hours before the application of benzoylpaeoniflorin (BPF; 0–10 *μ*M) for 48 hours. The mRNA levels of COX-1 and COX-2 were determined by the Ct-value method and normalized by the mRNA level of GAPDH. Ten *μ*M of dexamethasone (Dex) served as positive control. Values were expressed as the fold of increase to basal reading (untreated culture). Data were expressed as mean ± SEM, where *n* = 4; ^*∗*^*p* < 0.05; ^*∗∗*^*p* < 0.01.

**Figure 5:**
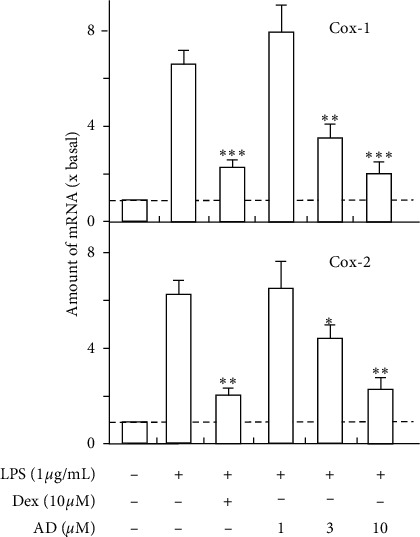
Amygdalin inhibits LPS-induced cyclooxygenase expression. Cultured HUVEC cells were pretreated with LPS (1 *μ*g/mL) for 3 hours before the application of amygdalin (AD; 0–10 *μ*M) for 48 hours. The mRNA levels of COX-1 and COX-2 were determined by the Ct-value method and normalized by the mRNA level of GAPDH. Ten *μ*M of dexamethasone (Dex) served as positive control. Values were expressed as the fold of increase to basal reading (untreated culture). Data were expressed as mean ± SEM, where *n* = 4; ^*∗*^*p* < 0.05; ^*∗∗*^*p* < 0.01; ^*∗∗∗*^*p* < 0.001.

Based on the above results, we further revealed the anti-inflammatory effects of active compounds. As expected, paeoniflorin could inhibit LPS-induced COX-1 and COX-2 mRNA expressions in a dose-dependent manner; the inhibition efficiency of 10 *μ*M paeoniflorin could reach ∼80% (Figure 3(a)). Besides, paeoniflorin could decrease the quantity of LPS-induced COX-2 protein in a dose-dependent manner. The effect of paeoniflorin was similar to that of dexamethasone, which was a positive control (Figure 3(b)). Benzoylpaeoniflorin inhibits LPS-induced COX-1 and COX-2 mRNA expressions in a dose-dependent manner, and the inhibition efficiency of 10 *μ*M could reach ∼60% (Figure 4). Amygdalin inhibits LPS-induced COX-1 and COX-2 mRNA expressions in a dose-dependent manner, and the inhibition efficiency of 10 *μ*M could reach ∼50% (Figure 5).

**Figure 6:**
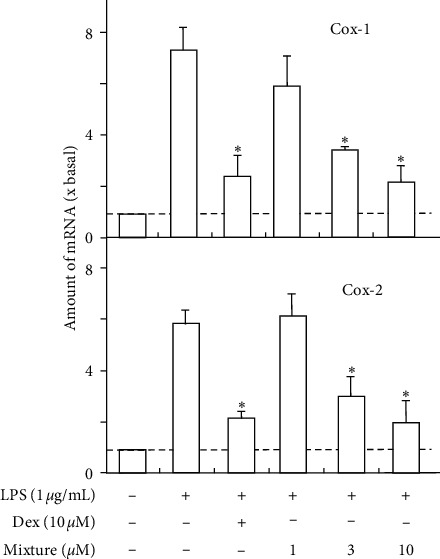
The effect of combination of three compounds in LPS-induced cyclooxygenase expression. Paeoniflorin, benzoylpaeoniflorin, and amygdalin were mixed in a ratio of 22 : 2:9 (refer to Table 1), as that in GFC, i.e., 10 *μ*g/mL GFC should contain 2.00 *μ*M paeoniflorin, 0.18 *μ*M benzoylpaeoniflorin, and 0.82 *μ*M amygdalin. Cultured HUVEC cells were pretreated with LPS (1 *μ*g/mL) for 3 hours before the application of the mixture (0–10 *μ*M) for 48 hours. The mRNA levels of COX-1 and COX-2 were determined by the Ct-value method and normalized by the mRNA level of GAPDH. Ten *μ*M of dexamethasone (Dex) served as positive control. Values were expressed as the fold of increase to basal reading (untreated culture). Data were expressed as mean ± SEM, where *n* = 4; ^*∗*^*p* < 0.05.

In order to evaluate the contribution of various ingredients to GFC, the combination of paeoniflorin, benzoylpaeoniflorin, and amygdalin was applied onto the cultures. The combined rate of three chemicals was in compliance with their concentration in GFC. The 10 *μ*g/mL of GFC should contain 2.00 *μ*M paeoniflorin, 0.18 *μ*M benzoylpaeoniflorin, and 0.82 *μ*M amygdalin. We mixed paeoniflorin, benzoylpaeoniflorin, and amygdalin equally. This mixture could suppress LPS-induced COX-1 and COX-2 mRNA levels in a dose-dependent manner (Figure 6). At 3.00 *μ*M, the effects of combination could march ∼80% of GFC's suppression at 10 *μ*g/mL. In other words, the contribution rate of three compounds to the anti-inflammatory functions of GFC reached at least ∼80%.

## 4. Discussion

GFC, a well-known herbal formula, is prescribed for women's diseases, e.g., dysmenorrhea, hysteromyoma, endometriosis, and other gynecological diseases [11]. The clinical effect of GCF is very remarkable to be used in China for over 2,000 years. The complexity of GFC of having five herbs hinders the discovery of functional mechanisms as a therapeutic agent in the disease treatment. In a previous study, a systematic approach to study herbal formulae has been successfully applied to reveal the chemical and biological assessments of Danggui Buxue Tang, Kaixin San, Fo Shou San, and Yu Ping Feng San [9, 12–14]. Therefore, these approaches were applied here in GFC to uncover the clinical treatment of dysmenorrhea. The anti-inflammatory effects of GFC had been confirmed to downregulate COX expression in HUVECs. The results were consistent with the previous animal studies [8, 15].

COX is a rate-limiting enzyme in the initial steps of prostaglandin synthesis and plays an important role during the synthesis of prostaglandins. COX-1 is distributed in the stomach, small intestine, kidney, and platelets, while COX-2, distributed only in the kidney and brain under normal circumstances, is induced by inflammation, colorectal tumor, and pain. COX-2 appeared to be involved mainly in early inflammatory processes, but COX-1 has no close relationship with the inflammation [16, 17]. Many kinds of inflammatory mediators including proinflammatory cytokines, oxygen-derived free radicals, and cells stimulate the body to produce an inflammatory response, which could induce COX-2 to express abundantly. Finally, the overexpression of COX-2 would induce synthesis and accumulation of prostaglandins and inflammatory cytokines in the damaged tissues, which subsequently produced a series of outcomes, such as local inflammation and tissue damage [18]. Paeoniflorin, benzoylpaeoniflorin, and amygdalin of GFC were shown to inhibit COX-2 expression in HUVECs. In parallel, paeoniflorin was shown to decrease COX-2 expression in the synovium, which was a benefit to suppress arthritis in a rat model [15, 19]. On the other hand, paeoniflorin could protect ischemia-induced brain damage by inhibiting COX-2-mediated activity in rats [20]. Overall, paeoniflorin is supposed to be a potential anti-inflammation chemical.

So far, which compound of GFC is responsible for the anti-inflammatory effect in HUVECs remains unclear and needs to be clarified in a further study. Based on Chinese Pharmacopoeia (2020), paeoniflorin, amygdalin, and paeonol are designated as markers for quantitative analysis of GFC by ultrasound-assisted extraction combined with HPLC-UV detection with isocratic elution. However, the cognition to active components in GFC still remains insufficient. We screened nine major components of GFC, and paeoniflorin, benzoylpaeoniflorin, and amygdalin were shown to contribute most of the effects of GFC in HUVECs. Benzoylpaeoniflorin, not registered in Chinese Pharmacopoeia, showed a positive effect; but its amount in GFC was much less than that of paeoniflorin and amygdalin. Therefore, the bioactivity results coincided well with the markers designated by Chinese Pharmacopoeia. Besides, GFC and its active components could be a complementary way to shed light on cardiovascular drug research.

## 5. Conclusions

Our study supports that GFC has a promising role in anti-dysmenorrhea function by decreasing COXs' expression. Besides, paeoniflorin, benzoylpaeoniflorin, and amygdalin could be considered as major regulators for the anti-dysmenorrhea effects of GFC.

## Figures and Tables

**Table 1:** Chemical and biological information of major compounds in GFC.

No.	Compound	Derivation	Content in GFC (%)^*a*^	Noncytotoxicity (*μ*M)	Bioactivity in HUVEC cells
1	Paeoniflorin	Paeoniae Radix Alba/Moutan Cortex	2.25	<1000	**+++**
2	Benzoylpaeoniflorin	Paeoniae Radix Alba/Moutan Cortex	0.28	<500	**++**
3	Amygdalin	Persicae Semen	2.15	<1000	**+**
4	Paeonol	Paeoniae Radix Alba/Moutan Cortex	1.43	<500	N
5	Cnnamic aldehyde	Cinnamomi Ramulus	0.20	<20	N
6	Cinnamic acid	Cinnamomi Ramulus	0.15	<1000	N
7	Pachymic acid	Poria	0.08	<25	N
8	Gallic acid	Moutan Cortex/Persicae Semen/Paeoniae Radix Alba	0.88	<250	N
9	Benzoic acid	Moutan Cortex/Paeoniae Radix Alba	0.12	<500	N

^a^The chemical content was cited from Zhang et al. (2015); the cytotoxicity data were expressed as the mean of *n* replicates (*n* ≧ 4). “+++” indicates that the inhibition ratio to the mRNA expression of COXs is lower than 70%. “++” indicates that the inhibition ratio is between 50% and 70%. “+” indicates that the inhibition ratio is between 30% and 50%. “N” indicates that the inhibition ratio is lower than 30%.

## Data Availability

All data generated or analyzed during this study are included in this published article.

## Abbreviations

COX: Cyclooxygenase
Dex: Dexamethasone
ECGS: Endothelial cell growth serum
GFC: Guizhi Fuling capsule
HUVECs: Human umbilical vein endothelial cells
LPS: Lipopolysaccharide
TCM: Traditional Chinese Medicine.
